# Extraction of useful bioisostere replacments from the PDB

**DOI:** 10.1186/1758-2946-3-S1-P37

**Published:** 2011-04-19

**Authors:** T Ritschel, DJ Wood, J de Vlieg, M Wagener

**Affiliations:** 1 Computational Drug Discovery Group, Center for Molecular and Biomolecular Informatics, Radboud University Nijmegen Medical Centre, Nijmegen, The Netherlands; 2 Molecular Design & Informatics, MSD, Oss, The Netherlands

## 

Bioisosteres are defined as structurally different molecules or substructures that can form similar intermolecular interactions, and therefore fragments that bind to similar protein pocket can be considered to have exhibited a degree of bioisosterism [1,2]. In this work a new method for the calculation of localized binding site similarities based on 3D-pharmacophore fingerprints is presented. The method needs no prior, time-consuming alignment of the proteins and therefore an on-the-fly searching of PDB scale crystal structure database for potential bioisosteric replacements is feasible. The binding site fingerprints are experimentally optimized to improve their performance. A variety of attributes of the fingerprints were considered in the optimization, including the placement of pharmacophore features, whether or not the fingerprints were fuzzified, and the resolution and complexity of the pharmacophore fingerprints (2-, 3- and 4-point fingerprints). Finally, fuzzy 3-point pharmacophore fingerprints were chosen to represent the optimal balance between computational resource requirements and the identification of potential replacements, and were therefore used to represent the localized binding sites in a searchable fragment database.

The utility of the approach is demonstrated by (i) separating known similar binding site pairs from random binding site pairs and (ii) a bioisosteric replacement study for fragments binding to subpockets of different proteins.
